# Gluten-specific antibodies of celiac disease gut plasma cells recognize long proteolytic fragments that typically harbor T-cell epitopes

**DOI:** 10.1038/srep25565

**Published:** 2016-05-05

**Authors:** Siri Dørum, Øyvind Steinsbø, Elin Bergseng, Magnus Ø. Arntzen, Gustavo A. de Souza, Ludvig M. Sollid

**Affiliations:** 1 Centre for Immune Regulation and Department of Immunology, University of Oslo and Oslo University Hospital - Rikshospitalet, 0424 Oslo, Norway; 2 Proteomics Core Facility, Oslo University Hospital-Rikshospitalet, 0424 Oslo, Norway; 3 Norwegian University of Life Sciences, NMBU, Department of Chemistry, Biotechnology and Food Science, 1432 Aas, Norway

## Abstract

This study aimed to identify proteolytic fragments of gluten proteins recognized by recombinant IgG1 monoclonal antibodies generated from single IgA plasma cells of celiac disease lesions. Peptides bound by monoclonal antibodies in complex gut-enzyme digests of gluten treated with the deamidating enzyme transglutaminase 2, were identified by mass spectrometry after antibody pull-down with protein G beads. The antibody bound peptides were long deamidated peptide fragments that contained the substrate recognition sequence of transglutaminase 2. Characteristically, the fragments contained epitopes with the sequence QPEQPFP and variants thereof in multiple copies, and they typically also harbored many different gluten T-cell epitopes. In the pull-down setting where antibodies were immobilized on a solid phase, peptide fragments with multivalent display of epitopes were targeted. This scenario resembles the situation of the B-cell receptor on the surface of B cells. Conceivably, B cells of celiac disease patients select gluten epitopes that are repeated multiple times in long peptide fragments generated by gut digestive enzymes. As the fragments also contain many different T-cell epitopes, this will lead to generation of strong antibody responses by effective presentation of several distinct T-cell epitopes and establishment of T-cell help to B cells.

Celiac disease is a chronic inflammatory enteropathy caused by ingestion of wheat gluten and similar proteins of barley and rye. The disease is considered mediated by T cells as there is a strong disease association with certain HLA-DQ allotypes, and as the patients have CD4+ T cells recognizing gluten peptides in the context of the disease associated HLA-DQ molecules^1^. The lesion of the small intestine is not characterized by massive CD4+  T cell infiltration, but rather by a huge increase in density of plasma cells^2^,^3^. Some of the infiltrating plasma cells secrete antibodies specific for gluten^4^,^5^. Whether and how gluten antibodies are involved in the immunopathogenesis of celiac disease is largely unknown. Case reports of patients successfully treated with B-cell depletion suggest that the humoral immune system plays an important role^6^,^7^.

The wheat gluten proteome is extremely complex and consists of several hundred different proteins of the glutenin (high and low molecular weight) and gliadin (α, γ, ω) varieties. In the gut, these proteins are enzymatically digested by endoproteases like pepsin, trypsin, chymotrypsin, elastase and carboxypetidase and then further broken down by exopeptidases of the brush border. The gluten proteins have similar amino acid sequences and often contain repeating stretches that are dominated by proline and glutamine residues. The high content of proline makes the gluten proteins resistant to extensive proteolysis^8^, and long fragments of gluten proteins survive in the upper part of the small bowel^9^ and can become exposed to the inductive part of the gut immune system as immunogenic peptides permitting responses by T cells and B cells. Many gluten-derived peptides are excellent substrates for the enzyme transglutaminase 2 (TG2), which can deamidate glutamine residues in certain sequence contexts and thereby convert them into glutamic acid. Interestingly, both the T-cell and B-cell response in celiac disease seem to be directed toward gluten peptides that have been deamidated by TG2^10^,^11^,^12^.

Antibodies do not only exert their function in extracellular fluids. Antibodies, as membrane bound immunoglobulins, also serve as the antigen receptors of B cells. Gluten-specific B cells could thus play a role as antigen presenting cells for gluten-specific T-cells. The requirement for this presentation is that the B- and T-cell epitopes are linked as part of a physical unit which can be taken up by the B-cell receptor for subsequent antigen processing and HLA mediated peptide presentation. The distribution of B-cell and T-cell epitopes in antigens is thus not random.

The epitopes recognized by gluten-specific CD4+ T cells in celiac disease are well characterized, not least through extensive testing with T-cell clones that represent monoclonal reporter reagents^13^. The gluten B-cell epitopes of celiac disease patients, however, until recently were only characterized by polyclonal reporter reagents, like serum antibodies^11^,^14^,^15^,^16^,^17^. Monoclonal reporter reagents recently became available by cloning and expression of antibodies from single IgA^+^ plasma cells from small intestinal biopsies of human subjects with untreated celiac disease^5^. Gluten-reactive IgA^+^ plasma cells were either identified after *in vitro* culture of single plasma cells from celiac lesions followed by ELISA screening for supernatant IgA with reactivity to chymotrypsin-digested and deamidated gliadin or by flow cytometry sorting of cells stained with two different synthetic gliadin peptides. The human monoclonal antibodies (hmAbs) obtained by these two approaches were specific to gliadin antigens, but showed reactivity to similar yet distinct synthetic gliadin peptides^5^. Thus, the procedures by which these hmAbs were generated would not identify which epitopes in the gluten proteome the hmAbs are primarily reactive with. This is obviously critical in order to elucidate the potential relationship between the B- and T-cell responses to gluten in celiac disease. Here, we report epitope mapping of gliadin-reactive hmAbs by antibody pull-down of fragments from complex proteolytic digests of gluten followed by sequencing of the isolated peptides by mass spectrometry.

## Methods

### Gliadin-reactive monoclonal antibodies

Gliadin-reactive hmAbs were produced as human IgG1 in HEK293A cells as previously reported^5^,^18^, and purified by protein G sepharose (GE Healthcare Life Sciences) affinity chromatography.

### Peptides

All peptides were purchased from GL Biochem Ltd, except for the γ-26mer peptide that was purchased from Peptide 2.0. The following peptides were used in the MALDI-TOF experiments: γ-gliadin 26mer; FLQPEQPFPEQPEQPYPEQPEQPFPQ, α-gliadin 33mer; LQLQPFPQPELPYPQPELPYPQPELPYPQPQPF and the short γ-gliadin peptide; PLQPEQPFP. In ELISA and AlphaLISA the following peptides were used: biotin-GSGSGSPLQPEQPFP, biotin-QPEQPFPEQPEQPEQPFPQPEQPFPWQPEQPFPQ, FLQPEQPFPEQPEQPYPEQPEQPFPQ, PQPQQPEQPFPQPQ, QQPFPQQPQQPYPQQPEQPFPQP, QPQQPFPQPEQPFPWQP, PQQPFPQPEQPFP, FPQPEQPFPWQP, PFPQPEQPFPWQPQQPFPQ, PQQPEQPFP, biotin-GSGSGSPQQPQQPFP, biotin-GSGSGSPQQPEQPFP, biotin-GSGSGSPQQPQQQFP, biotin-GSGSGSPQQPEQQFP, biotin-GSGSGSPQQPQQSFP, biotin-GSGSGSPQQPEQSFP, biotin-GSGSGSPQQPQQTFP, and biotin-GSGSGSPQQPEQTFP.

### Preparation of gliadin digest treated with TG2

Wheat gliadin was digested with proteolytic enzymes as previously described^19^. In short, gliadin extracted from grocery store wheat flour (Møllerens) was digested with chymotrypsin (Sigma) at 200:1 (w:w) in 0.1 M NH_4_HCO_3_ with 2 M urea at 37 °C for 24 h followed by enzyme inactivation for 5 min at 95 °C. Chymotrypsinated gliadin (0.5 g) was dissolved in 0.01 M acetic acid (adjusted to pH 1.8 with HCl) and incubated with pepsin (1:100, Sigma) for 3.5 hours at 37 °C, and 10 min at 95 °C. After cooling and pH adjustment to 7.8 (with NaOH), trypsin (1:100, Sigma) was added and incubated overnight at 37 °C, followed by enzyme inactivation for 10 min at 95 °C. The sample was further digested with elastase (1:500, Sigma) and carboxypeptidase A (1:100, Sigma) for 2 h at 37 °C shaking, followed by enzyme inactivation for 10 min at 95 °C. The gliadin digest was dialyzed (MWCO: 1000) and freeze dried.

The PTCEC (pepsin, trypsin, chymotrypsin, elastase, carboxipeptidase) gliadin digest (~5 mg) was next fractionated by size exclusion chromatography using an Äkta Purifier system (Äkta purifier 10, GE Healthcare Life Sciences) with a Superdex peptide 10/300 GL column (GE Healthcare Life Sciences, L × I.D: 30 cm × 10 mm, 13 μm particle size). A flow rate of 0.5 ml/min was applied using Milli-Q water as mobile phase, and fractions of 0.5 ml were collected and speed-vac’ed. The fractions were reconstituted in 60 μl Milli-Q water and two to three adjacent fractions were pooled.

The pooled fractions were treated with 0.1 μg/μl TG2 in 100 mM Tris-HCl pH 7.4 supplemented with 2 mM CaCl_2_ for 60 min at 37 °C, and the reaction was terminated by adding iodoacetamide (2 mM final concentration). Recombinant TG2 with an N-terminal hexa-histidine tag was expressed in *E. coli* and purified as previously reported^20^.

The gliadin fractions treated with TG2 (termed TG2-gliadin) were subjected to pull-down with hmAbs. Fractions containing the highest molecular weight peptides were not used in the pull-down experiments.

### Antibody pull-down

Each hmAb (40 μg, 260 pmol) was incubated with pooled fractions of TG2-gliadin or with synthetic peptides (1500 pmol) for 20 minutes at room temperature (RT). Dynabeads Protein G (Life Technologies) were added to the samples and incubated for 20 minutes at RT (125 μg beads/μg IgG). After washing with 5 × 500 μl PBS/0.1% octylglucoside, bound peptides were eluted with 0.1% TFA for 10 min at RT. A rotavirus specific hmAb (Rota-2B04) was used as negative control^21^.

### Mass spectrometry analysis

Peptides were analyzed by MALDI-TOF MS or by nano-LC-MS/MS. MALDI-TOF-MS (Ultraflex II, Bruker Daltonics) was used for analysis of low complexity samples which were spotted onto stainless-steel target plates using a matrix of 10 mg/ml α-cyano-4-hydroxycinnamic acid in 70% acetonitrile (ACN)/0.1% trifluoroacetic acid (TFA). Spectra were recorded in the positive and reflector mode. More complex samples were analysed by nano-LC-MS/MS using a Q Exactive hybrid quadropole-orbitrap mass spectrometer with an EASY-spray ion source (both from Thermo Fisher Scientific) that was coupled to either of two liquid chromatography systems, nano-HPLC (Dionex Ultimate 3000 nano-LC system; NCS-3500RS NANO) or EASY-nLC 1000 (Thermo Fisher Scientific). Two different set-ups were used. 1) In the Dionex nLC set-up, samples were loaded onto a trap column (C18, 100 μm × 2 cm, PepMap RSLC, Thermo Fisher Scientific) before separation on an EASY-Spray column (C18, 25 cm × 75 μm ID, 2 μm particles, Thermo Fisher Scientific) using a binary gradient consisting of solvent A (0.1% formic acid (FA) and solvent B 90% ACN/0.1% FA) at a flow rate of 0.3 μl/min. The Q Exactive instrument was operated in data-dependent acquisition mode to switch automatically between orbitrap-MS and higher-energy collisional dissociation (HCD) orbitrap-MS/MS acquisition using the Xcalibur 2.2 software. Single MS full-scan in the orbitrap (300–1750 m/z, 70000 resolution, ACG target 3e6, maximum IT 50 ms) was followed by 10 data dependent MS/MS scans in the orbitrap after accumulation of 2e05 ions in the C-trap or an injection time of 100 ms (fixed injection method) at 17500 resolution (isolation 3.0 m/z, underfill ratio 10%, dynamic exclusion 45 s) at 25% normalized collision energy. 2) In the EASY-nLC set-up, samples were separated on the same EASY-Spray column using a binary gradient consisting of solvent A (0.1% FA) and solvent B (ACN/0.1% FA) at a flow rate of 0.3 μl/min. The Q Exactive instrument was operated in data-dependent acquisition mode where single MS full-scan in the orbitrap (400–1200 m/z, 70000 resolution, ACG target 3e6, maximum IT 100 ms) was followed by 10 data dependent MS/MS scans in the orbitrap after accumulation of 1e05 ions in the C-trap or an injection time of 100 ms (fixed injection method) at 17500 resolution (isolation 3.0 m/z, underfill ratio 0.1%, dynamic exclusion 30 s) at 25% normalized collision energy. Obtained data are deposited to the PRIDE partner repository^22^ (dataset identifier PXD002678).

### Database search

LC-MS/MS data were analyzed with the software MaxQuant version 1.5.1.2 23 using the search engine Andromeda^24^ to search against a *Triticum aestivum* database (total of 4722 entries) extracted from the UniprotKB database release September 2013 (European Bioinformatics Institute). In all searches the enzyme specificity was set as none and the variable modifications pyro-glu (N-term Q), deamidation (NQ) and oxidation (M) were selected. For analysis of Q Exactive data, the mass error tolerance for MS scans was first searched with an error window of 20 ppm and then with a main search error of 6 ppm. Mass tolerance for MS/MS scans was set to 20 ppm. A false discovery rate of 1% and a PEP score of 0.1 were used. A few peptides pulled down with the negative control hmAb were removed from the list of enriched peptides. In addition, a couple of peptides that derived from actin and other non-gluten proteins were identified (Supplementary Table S1).

### Peptide sequence analysis

To address the epitope specificities of the gliadin-specific hmAbs, a script in Python (version 2.7.6) was made to count frequencies of different motifs among the eluted sequences. The script calculated all possible sequence patterns with length between 3–15 residues by character iteration and substring generation. The frequency of each pattern was calculated and graphic outputs were generated. For some hmAbs, the frequency of similar but not identical peptide motifs was assessed. The most common 7mer motif was first identified and this was used to reanalyze the sequences with the webware NPS@ (Network Protein Sequence Analysis, Pôle Bioinformatic Lyonnais) using the “PATTINPROT search” allowing for disparity at one or two positions. WebLOGO 3.4 (http://weblogo.berkeley.edu) was used for sequence visualization.

### ELISA reactivity of gliadin-reactive hmAbs to synthetic gliadin peptides

Biotinylated synthetic gliadin peptides (500 nM) were used as antigens in streptavidin coated ELISA plates (Nunc, 436014). Gliadin-specific hmAb 1130-3B01 or 1130-3A02 at a concentration of 2 μg/ml and with fourfold dilution were used to generate titration curves. The ELISA was run with alkaline phosphatase conjugated anti-human IgG (Southern Biotech 9040-04) at 1:4000 dilution, phosphatase substrate (Sigma S0942-200TAB) and absorbance measured in a plate reader at 405 nm (Multiskan Ascent 96, Thermo Fischer Scientific). PBS pH 7.4 was used as buffer, and the plates were washed three times with PBS with 0.05% Tween between each step.

### AlphaLISA assays

The relative affinity of three hmAbs (1002-1E01, 1002-1E03 and 1130-3B01) to a panel of gliadin peptides was investigated using an AlphaLISA platform. AlphaLISA acceptor beads (Perkin Elmer, Unconjugated AlphaLISA^TM^ Acceptor beads, 6672001) were coated with anti-human IgG (Dako, Polyclonal Rabbit Anti-Human IgG, A0423) according to manufacturers’ protocol (Perkin Elmer, *Antibody Conjugation to AlphaLISA^®^ Acceptor Beads Detailed Protocol*). The anti-IgG AlphaLISA beads 6 μg/ml and hmAb 0.5 μg/ml were mixed and incubated for 1 hour at RT in dark. Then, 15 μl of the solution was transferred to each well in a 384-well AlphaLISA plate (Perkin Elmer, 384-well OptiPlate, 6007290), together with 5 μl analyte consisting of 40 nM biotinylated PLQPEQPFP peptide and diluting concentrations of non-biotinylated competing peptides (5 mM, 1:3 dilution). After incubation for 1 hour at RT in dark, 15 μl of streptavidin coated Alphascreen donor beads (24 μg/ml) (Perkin Elmer, AlphaScreen^®^ Streptavidin Donor beads, 6760002S) were transferred per well, and the plate incubated for 1 hour at RT in dark. AlphaLISA Signal was measured with Envision 2104 Multilabel Plate Reader (Perkin Elmer). PBS pH 7.4 and 0.1% Puviol was used as buffer. The binding of whole IgG1 versus Fab of gliadin-reactive antibody to gliadin peptide was investigated in a similar competitive AlphaLISA assay. Here we used diluting titrations of IgG1 or Fab of hmAb 1002-1E03 together with either AlphaLISA acceptor beads conjugated with hmAb 1002-1E03 and biotinylated 34mer ω-gliadin (2.5 nM), or AlphaLISA acceptor beads conjugated with PLQPEQPFP together with biotinylated hmAb 1002-1E03 (0.1 mg/ml).

In experiments addressing epitope mulitivalency, AlphaLISA Acceptor beads were coated with the gliadin-specific hmAb 1002-1E03. Dilutions of synthetic peptides with monovalent or multivalent display of the QPEQPFP epitope, or hmAb and F(ab2) was used as competitors.

## Results

### Antibody pull-down and identification of peptides by mass spectrometry

A method was established to identify the preferred peptide epitopes of gliadin-specific hmAbs. The hmAbs were incubated with size-separated fractions (Supplementary Figure 1) of TG2-gliadin (enzymatically digested and TG2-treated gliadin) and hmAb-peptide complexes were isolated with magnetic protein G beads. Subsequently, the antibody bound peptides were eluted from the protein G beads and the eluted peptides were analyzed using a Q Exactive mass spectrometer (Supplementary Figure 2). The TG2-gliadin fractions were compared pre and post pull-down. To control for unspecific binding, a rotavirus-specific hmAb was included as a negative control. Of the thirteen gliadin-reactive hmAbs tested, nine derived from IgA^+^ plasma cells isolated by flow cytometry with two different synthetic gliadin peptides used for staining (biotin-GSGSGS-PLQPEQPFP, PLQPEQPFP for short; biotin-(PEG)-LQLQPFPQPELPYPQPELPYPQPELPYPQPQPF, deamidated 33mer for short) and four hmAbs derived from *in vitro* cultured plasma cells secreting IgA reactive to complex deamidated gliadin (Table 1).

For eleven of the thirteen hmAbs, several unique gluten peptides (number of peptides identified by a single hmAb ranging from 19–552 peptides) were identified in the pull-down analyses (Table 1, Supplementary Table S1). One hmAb (1065-4G05) did not enrich detectable peptides, and only four peptides were identified in the pull-down analyses from the last hmAb (1065-4C01). This could possibly be explained by low affinity of the hmAbs, as observed in ELISA (data not shown). These hmAbs were hence excluded from further analyses.

The enzymatic digest of gliadin used for the antibody pull-down contained glutenin peptides, thus probing would also be towards this part of the gluten proteome. In general, peptide fragments from α-, γ-, ω-gliadins and low-molecular weight glutenins were pulled down while fragments of α-gliadins were relatively infrequent.

### Enrichment of long deamidated peptide fragments

**Figure 1:**
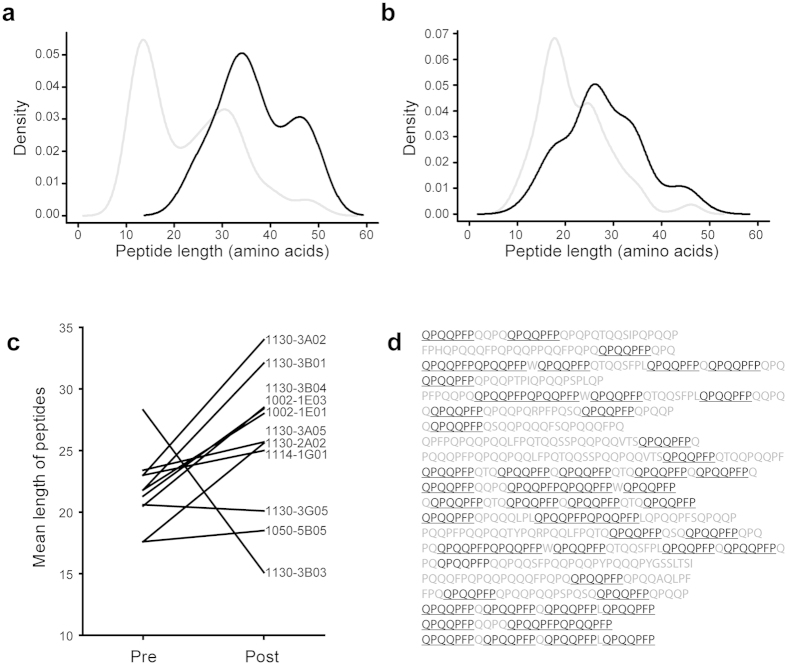
The hmAbs pull-down long peptides with repeated motifs. (**a**) Kernel density plot of the peptide length identified by Q Exactive mass spectrometry in TG2-gliadin fractions pre (grey line) and post (black line) pull-down by the hmAbs 1130-3A02 (**b**) and 1002-1E01. (**c**) Mean peptide length pre and post pull-down from TG2-gliadin fractions with all hmAbs. (**d**) Peptides pulled down with hmAb 1130-3A02. The most frequent 7mer motif is shown in black underlined.

The peptides present in post pull-down samples were generally longer than peptides in the pre pull-down samples. This pattern, as exemplified by the hmAb 1130-3A02 and hmAb 1002-1E01 (Fig. 1a,b), was present for all but two (1130-3B03 and 1130-3G05) of the antibodies (Fig. 1c). Many of the enriched peptide fragments harbored repeated sequence motifs (Fig. 1d). We further observed that the large majority of the identified hmAb-enriched gluten peptides had been deamidated by TG2 (Table 1). As expected, most of these peptides (64% of 1800) were deamidated in the QXP motif, which is in keeping with the reported sequence specificity of TG2^25^,^26^. However, in some peptides the exact deamidation sites could not be unambiguously determined by the MaxQuant search engine. Gluten proteins and peptides are peculiar in that they contain repeats consisting of proline residues and multiple sequential Q residues. This results in many short fragments of proline and glutamine repeats with identical masses in the MS fragment spectra, which hinders exact sequence assignment of deamidation sites, particularly in cases of partial fragmentation. Many gluten peptides were reported by MaxQuant to be deamidated at Q2 in the frequent sequence motif QQP (Supplementary table 1). However, TG2 does not deamidate a Q with a P in position +1, and it prefers to deamidate Q residues in the QXP motif. This suggests along with the partial fragmentation of the MS spectra that it is Q1 in the QQP motifs that are deamidated in these particular peptides. Because of the uncertainty in determining deamidation sites for some peptides, we used the native sequence of all peptides in the analyses addressing common sequence motifs.

### Enriched peptides are not necessarily similar to peptides to which the hmAbs were selected

For some of the hmAbs, the enriched peptides had different peptide sequences than the selecting peptide antigen originally used to isolate the IgA^+^ plasma cell (Table 1). This was particularly observed for the hmAbs of plasma cells sorted with the α-gliadin 33mer peptide LQLQPFPQPELPYPQPELPYPQPELPYPQPQPF (i.e. mAbs 1130-3B04, 1130-3A02, 1130-3B01, 1130-3A05, 1130-3B03, 1130-3G05).

### The enriched peptide fragments share motifs

**Figure 2:**
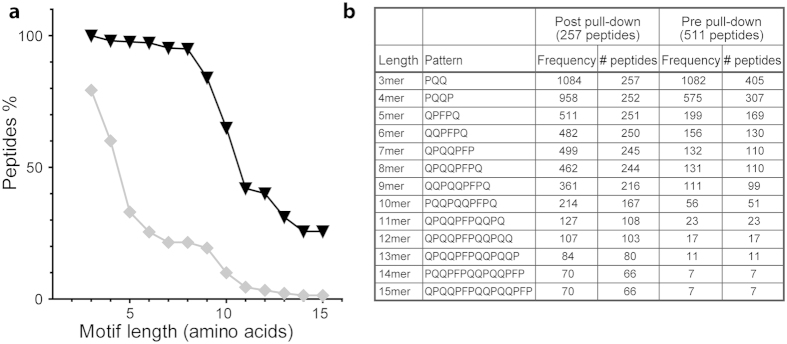
Common motifs in peptides pulled down by hmAb 1130-3B04. (**a**) Percent of peptides sharing identical sequence motifs, of 3 to 15 residues in length, post (triangles/black line) and pre pull-down (diamonds/grey line) from a fraction of TG2-gliadin by the hmAb 1130-3B04. (**b**) Sequence motifs and the frequency and number of peptides harboring the motifs.

The observation of repeated motifs in the pulled down peptides, prompted us to search for sequences of 3–15 residues common to all peptide fragments pulled down by the individual hmAbs. Most peptide fragments harbored a shared motif until a certain length. Beyond this length the frequency of peptides carrying the shared motif dropped dramatically. The results for hmAb 1130-3B04 are shown as an example, demonstrating that for this hmAb most fragments carried a shared motif of eight residues or shorter (Fig. 2a) that typically were extensions of a core sequence (Fig. 2b). On this basis, we explored for the presence of 7mer motifs for all the hmAbs. For the hmAb 1130-3B04 as well for the hmAbs 1002-1E01, 1130-3A02, 1130-2A02, 1002-1E03 and 1130-3A05 the most frequent 7mer motif was the sequence QPQQPFP. More than 88% of the peptides pulled down with these hmAbs harbored this motif (Table 1).

### Some hmAbs allow variation in their target motifs

**Figure 3:**
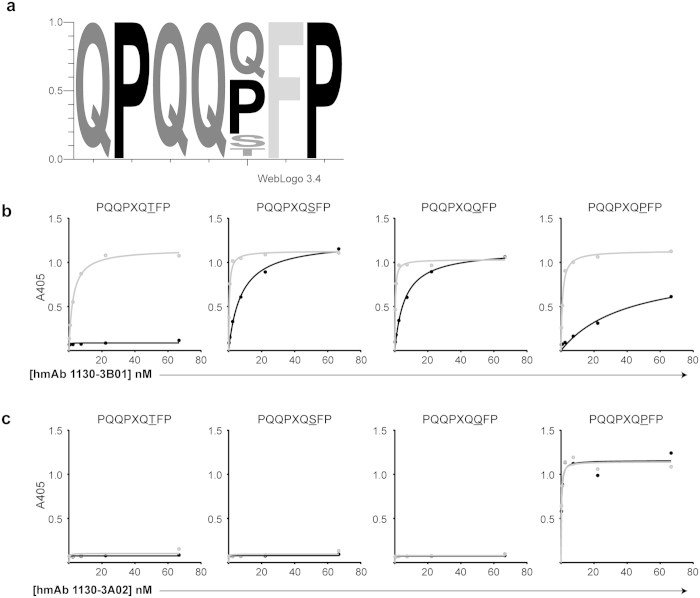
Sequence motif of peptides pulled down by hmAb 1130-3B01. (**a**) Sequence motif in peptides pulled-down with hmAb 1130-3B01, based on 85% similarity with most common 7mer motif QPQQQFP, as generated by WebLOGO 3.4. (**b**) ELISA reactivity of the hmAbs 1130-3B01 (**c**) and 1130-3A02 to synthetic gliadin peptides (native sequences in black and deamidated sequences in grey). Different antibody concentrations were used to generate affinity curves, as indicated on the x-axis.

For hmAbs 1114-1G01, 1130-3B01, 1050-5B05, 1130-3B03 and 1130-3G05, the frequencies of the most common 7mer motifs were clearly lower than for the other hmAbs. This could be because these hmAbs reacted with peptides with similar, but not necessarily identical sequences. We thus used “Pattinprot” (PBIL.ibcp.fr) to check for presence of 7mer motifs allowing for one or two amino acid variations in the QPQQPFP motif (85% or 60% similarity). This resulted in identification of motifs present at high frequencies for 1130-3B01, 1050-5B05 and 1114-1G01 (Table 1). The most common 7mer motifs for the hmAbs 1050-5B05 and 1114-1G01 were present in 44% and 73% of the identified peptides, which following the “Pattinprot” analysis increased to 83% and 100% by allowing for variation at positions 1 and 7 of the QPQQPFP motif (Table 1). The results for the hmAb 1130-3B01, shown by a sequence logo representation (Fig. 3a), indicated sharing of the motif QPQQXFP (X = P, S, T, Q). Reactivity to this motif was verified by ELISA which also revealed preferential reactivity to the deamidated versions of the peptides (Fig. 3b). By contrast, the hmAb 1130-3A02 recognized equally well native and deamidated version of the peptide containing the sequence QPQQPFP, but this hmAb had no reactivity to other peptides that were reactive with hmAb 1130-3B01 (Fig. 3c). This illustrates that there are differences in epitope fine specificity including the involvement of the glutamate residue between different hmAbs.

### Sequences flanking the target motif may influence the affinity

**Figure 4:**
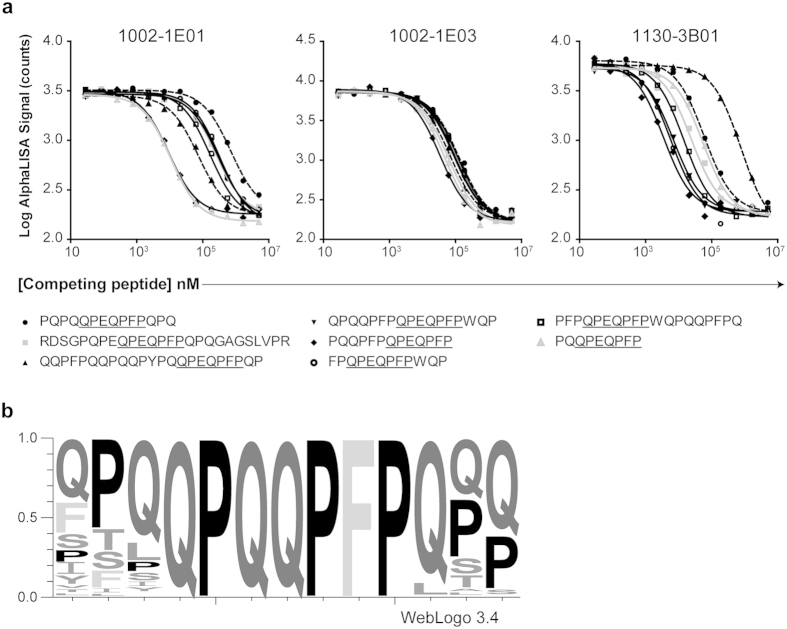
Binding affinity of hmAbs to QPEQPFP-containing peptides depends on residues flanking the motif. (**a**) AlphaLISA affinity of the hmAbs 1002-1E01, 1002-1E03 and 1130-3B01 to PLQPEQPFP and the competitive effect of a panel of different synthetic gliadin peptides harboring the QPEQPFP sequence motif at different concentrations (nM) as indicated on the x-axis. (**b**) Sequence motif obtained by searching a *Triticum aestivum* database with “Pattinprot” using the motif XXXQPQQPFPXXX (X = any amino acid). Sequence logo generated by WebLOGO 3.4.

To further understand the specificity of the anti-gluten antibodies, we tested hmAbs 1002-1E01, 1002-1E03 and 1130-3B01 for reactivity to a panel of deamidated synthetic gliadin peptides harboring the key sequence QPEQPFP (Q → E substitution in position 3) in a competitive AlphaLISA assay (Fig. 4a). While 1002-1E03 showed similar affinity for all peptides in the panel, the affinity of 1002-1E01 and 1130-3B01 to the different peptides varied by 1-2 logs. This demonstrates that although a “dominant” motif is found for the majority of the gliadin-reactive hmAbs, the flanking regions of the sequence motif will affect the binding affinity. In the gluten proteins, the QPQQPFP sequence motif can be found with a variety of different amino acids in the flanking regions (Fig. 4b).

### Epitope multivalency and pull-down enrichment

**Figure 5:**
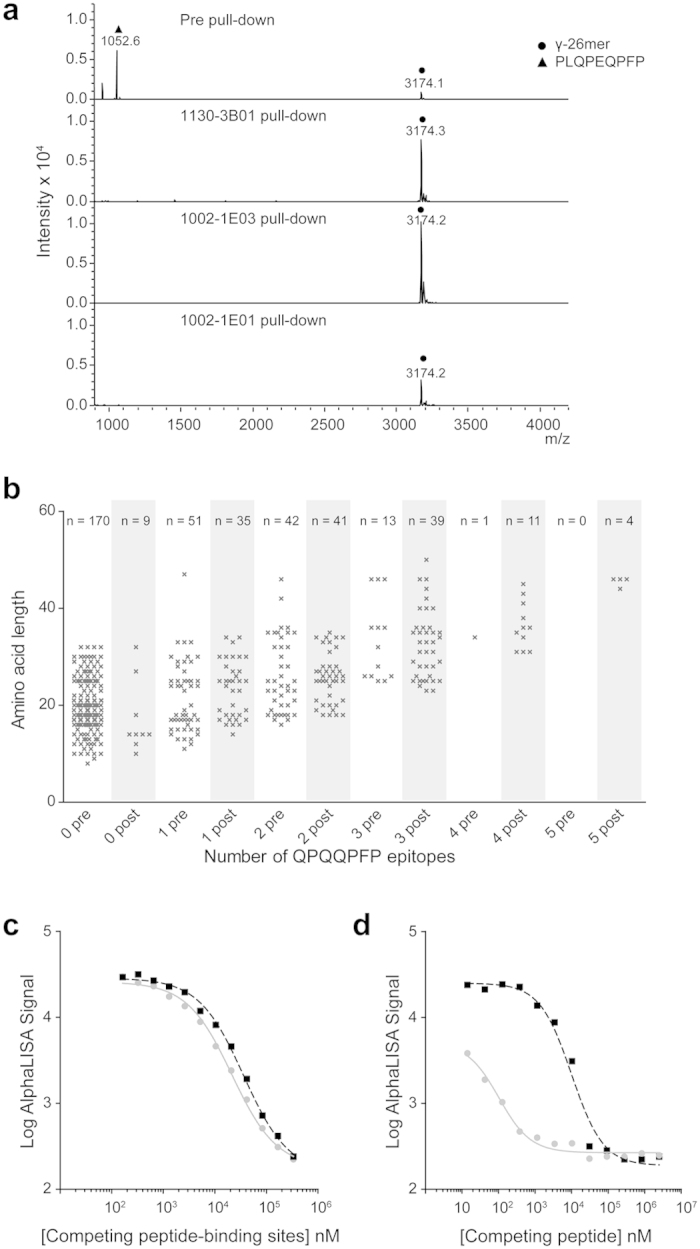
hmAbs show better reactivity to gluten peptides with repeats of epitopes. (**a**) Pull-down with the hmAb 1130-3B01, 1002-1E03 or 1002-1E01 from samples with equimolar amounts of the PLQPEQPF peptide and the γ-gliadin 26mer peptide. MALDI-TOF mass spectra of pre (upper panel) and post (three lower panels) samples are depicted. (**b**) Pull-down with hmAb 1002-1E03 from a size fraction of a gliadin digest treated with TG2 demonstrating that the hmAb preferentially pull-down long peptides with multiple repeats of epitopes. The number of QPQQPFP epitopes found in each peptide fragment and the length of the fragments in samples pre (grey) and post pull-down (black) are shown. Each cross represents one peptide fragment, and the numbers of unique peptide fragments with the different number of epitopes are given on top. (**c**) AlphaLISA competition assay comparing the relative binding of bead-conjugated hmAb 1002-1E03 to the soluble 34mer ω-peptide in the presence of competing soluble whole antibody (grey solid line) or Fab fragment (black dashed line) of the hmAb 1002-1E03. (**d**) Inhibition of binding of bead-conjugated hmAb 1002-1E03 to soluble PLQPEQPFP by FLQPEQPFPEQPEQPYPEQPEQPFPQ (grey solid line) or PLQPEQPFP (black dashed line).

To further scrutinize the enrichment for long peptides with repeated sequence motifs, we incubated a synthetic peptide mix containing equimolar amounts of the deamidated γ-gliadin peptide PLQPEQPFP (epitope x1 underlined) and the longer 26mer γ-gliadin peptide FLQPEQPFPEQPEQPYPEQPEQPFPQ (epitope x2 underlined) with the hmAbs 1130-3B01, 1002-1E03 and 1002-1E01 before incubation and pull-down with protein G beads, peptide elution and MALDI-TOF MS peptide detection (Fig. 5a). For all hmAbs the long peptide was the only detectable species in the pull-downs. Looking at epitope distribution in peptides of the gliadin digests pre and post pull-down, it was striking that longer peptides with multiple copies of the epitopes were pulled down (Fig. 5b). This suggested that the enrichment for long fragments with repeated motifs could be explained by more efficient binding of peptides harboring multiple epitopes. When comparing in a competitive AlphaLISA the binding of intact IgG1 vs Fab fragment of the hmAb 1002-1E03 for binding to the ω-gliadin peptide QPEQPFPEQPEQPEQPFPQPEQPFPWQPEQPFPQ (epitope underlined), equal binding was observed (Fig. 5c) suggesting the same binding affinity of the Fab and the intact bivalent antibody for binding of this peptide in solution. However, when the hmAb 1002-1E03 was immobilized on beads and binding of the deamidated γ-gliadin peptide PLQPEQPFP and the longer 26mer γ-gliadin peptide FLQPEQPFPEQPEQPYPEQPEQPFPQ (epitope underlined) was compared, the longer peptide bound substantially better (Fig. 5d) suggesting that in this setting the longer peptide with its two repeated epitopes allowed for increase in binding avidity.

### Other factors influencing the peptide pull-down

Qualitative and quantitative aspects of the gliadin fractions from which peptides in a competitive fashion were pulled down could influence which peptides were identified. To investigate influence of qualitative aspects, the hmAb 1130-3B01 was incubated with a synthetic peptide mix containing equimolar amounts of the deamidated α-gliadin 33mer peptide and the peptide PLQPEQPFP, which contains the deamidated QPQQPFP 7mer motif. The hmAb-peptide complexes were isolated, and bound peptides were analyzed by MALDI-TOF MS (Supplementary Fig. 3a). Only the α-gliadin 33mer peptide was enriched by the hmAb. In contrast, when incubating the hmAb with an equimolar mix of the deamidated α-gliadin 33mer peptide and a deamidated γ-gliadin 26mer peptide harboring the QPEQPFP motif in two copies (FLQPEQPFPEQPEQPYPEQPEQPFPQ), only the deamidated γ-gliadin 26mer peptide was pulled down (Supplementary Fig. 3b). This suggested that the hmAb preferred to bind long peptides harboring multiple copies of the QPEQPFP motif, and if present like in the competitive environment in the gliadin fractions, these peptides would dominate in the pull-down.

Quantitative aspects could also influence peptide pull-down as potential target peptide sequences were not present at equal concentrations in the gel filtration fractions. A substantial proportion of all peptides (21–39%) in the pre pull-down fractions harbored the QPQQPFP motif reflecting dominance of fragments from ω-gliadin proteins (about 15%), γ-gliadin proteins (about 45%) and low-molecular weight glutenin proteins (about 25%). In contrast, only 1.5–3% of the identified peptides harbored the typical PQPQLPY α-gliadin motif, and about 12% of the fragments were derived from α-gliadin proteins.

Of the six hmAbs from IgA + plasma cells sorted with the deamidated α-gliadin 33mer peptide, four of them were reactive to the PLQPEQPFP peptide in ELISA and AlphaLISA. Two of the hmAbs, 1130-3B03 and 1130-3G05, showed no reactivity to PLQPEQPFP (Table 1). Notably, no shared motifs were found among the peptides pulled down with these two hmAbs. Of the 19 peptides enriched by hmAb 1130-3B03, only one single peptide (LQLQPFPQPQLPYPQPHLPYPQPQP, see Supplementary Table S1) shared a part of its sequence with the α-gliadin 33mer peptide. To investigate whether this could relate to the low abundance of α-gliadin 33mer peptide in the gliadin fractions, we performed a pull-down experiment in a peptide mixture with equimolar amounts of the synthetic γ-gliadin 26mer and the α-gliadin 33mer peptide using hmAb 1130-3B03. The hmAb-bound peptides were analyzed by MS. MALDI-TOF spectra pre and post 1130-3B03 pull-down, demonstrated a preferential enrichment of the α-gliadin 33mer peptide (Supplementary Figure 3c). This suggests that the α-gliadin 33mer peptide is a good antigen for 1130-3B03, although it could not be readily identified in the post pull-down from the gliadin fraction. This could relate to the antibody affinity to the α-gliadin 33mer relative to other gliadin peptides, and the concentration of this peptide in the pre pull-down fraction.

### The hmAb-enriched peptide fragments harbor several different gluten T-cell epitopes

Potentially, gluten-specific B cells could serve an important role by presenting antigen to gluten-specific CD4 + T cells. We thus searched for the presence of gluten T-cell epitopes in the identified gluten peptides pre and post hmAb pull-down. As shown in Table 2, we found that more than 80% of all peptides pulled down with the six hmAbs 1002-1E01, 1130-3B04, 1130-3B01, 1002-1E03, 1130-3A02 and 1130-2A02, contained known gluten T-cell epitopes. The gluten T-cell epitopes DQ2.5-glia-y4c (QQPQQPFPQ) and/or the DQ2.5-glia-y5 epitope (QQPFPQQPQ) were the most frequent and were found in up to 84% and 60% of the enriched peptides, respectively.

**Figure 6:**
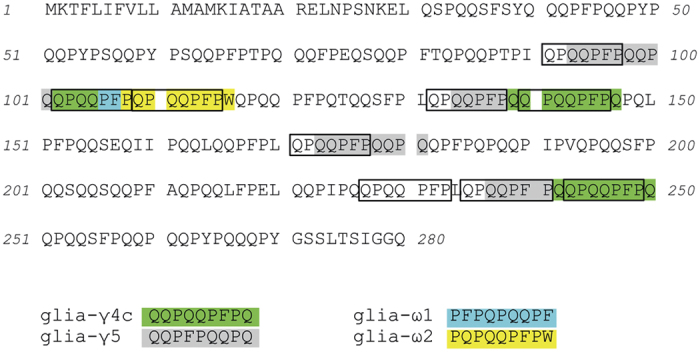
Co-localization of gliadin T-cell and B-cell epitopes in an ω-gliadin protein (accession number: Q9FUW7). The 9mer core sequences of known T-cell epitopes recognized by HLA-DQ restricted CD4 + T cells in celiac disease^13^ are highlighted in different colors. The hmAb epitope QPQQPFP is framed. Of note, the sequence of the native protein is given and glutamine (Q) residues that are targeted by TG2 for modification to glutamic acid are not marked.

B- and T-cell epitopes in gluten proteins appear to be in close proximity or overlap^11^,^14^. Our results confirm this notion. The hmAb binding motif QPQQPFP and the T-cell epitopes are most often overlapping in the gliadin proteins. This is particularly striking in the ω-gliadin protein (Accession number: Q9FUW7) visualized in Fig. 6. In this protein, 9 copies of the 7mer motif are present. All copies, except one, are overlapping with one or more T-cell epitopes. The DQ2.5-glia-γ5 epitope overlaps with four copies of the binding motif, DQ2.5-glia-γ4c overlaps with three copies, while DQ2.5-glia-ω1 and DQ2.5-glia-ω2 both overlap with one copy.

The T-cell epitope containing peptides were not present at equal concentrations in the gluten fractions before pull-down. While a substantial part of the peptides harbored γ-gliadin T-cell epitopes, only a few peptides harbored ω-gliadin T-cell epitopes. Thus when comparing the identified peptides pre and post hmAb pull-down, a massive enrichment was observed for both γ-gliadin and ω-gliadin T-cell epitopes.

## Discussion

We have characterized the natural binding targets of eleven gluten-specific hmAbs made by expression cloning of antibody genes of single intestinal IgA^+^ plasma cells from celiac disease patients. The natural binding targets of hmAbs were identified by isolating and sequencing a large number of fragments pulled down from fractions of gluten (gliadin) that had been treated with digestive enzymes and TG2. The majority of the hmAbs were established from staining plasma cells with labeled synthetic peptides. In several instances, the hmAbs selected for gluten peptides which differed from the selecting peptides.

Several interesting observations emerge from our experiments. The most interesting finding was that the hmAbs pulled down long peptide fragments of γ-gliadins, ω-gliadins and low molecular weight glutenins that all harbored repeated motifs. For the majority of the hmAbs (11 of 13), this type of motifs could be identified. The motifs all contained a short PQQ sequence, but they differed by a few variations in the flanking residues. While the majority of the hmAbs pulled down peptides that shared the QPQQPFP motif, some of the hmAbs were more promiscuous and enriched for peptides that harbored up to four different amino acids in certain positions of the 7mer motif. Testing different peptides with the same sequence core (QPEQPFP), but with various flanking regions in a competitive AlphaLISA assay, revealed that the antibodies’ affinity for the different peptides varied. These results suggest that the antibody response to gluten in celiac disease is generated in response to a few immunodominant epitopes, typically displayed in repeats, with variation in fine specificity between individual antibodies.

The enrichment for long fragments with repeated motifs likely relate to epitope multivalency. This enrichment was observed in experimental settings where the multivalent peptide fragments could engage more than one antibody molecule. This scenario would mimic the situation at the surface of a B cell where a multivalent antigen would be able to engage several B-cell receptors on the cell surface, followed by B-cell receptor crosslinking and B-cell activation. This could be a major reason why the B-cell epitopes in gluten proteins are sequence motifs which have multivalent display within long proteolytically resistant fragments. Final proving of this notion will require extensive testing of live B cells with B-cell receptors, preferably primary naïve B-cells, as has been done for other linear peptide epitopes and haptens in models of genetically modified animals^27^,^28^.

The peptide fragments pulled down by the hmAbs typically contained glutamate residues introduced by TG2-mediated deamidation. Further, in general, there was an enrichment of deamidated peptides when comparing pre and post pull-down samples. This was the case even with hmAbs that did not distinguish between synthetic peptides in native and deamidated versions in ELISA. The reason for this is that the QPQQPFP motif contains the QXP motif typically targeted by TG2^25^,^26^, and the hmAbs would react with deamidated peptides in the TG2-treated digests even though the glutamate residue is not necessarily part of the epitope.

The QPEQPFP motif has been described as immunodominant in several serological studies investigating IgG and IgA reactivity to gliadin^11^,^16^,^17^. However, the previous studies were investigating polyclonal serum reactivity to short synthetic gluten peptides. Here we show that this motif seems to be the primary epitope in the gluten proteome also for the IgA antibodies of plasma cells in the celiac disease intestinal lesion.

The gluten-specific hmAbs typically pulled down peptides with multiple gluten T-cell epitopes, where the hmAb binding motif and the T-cell epitopes overlapped or were in close proximity. This argues for a role for gluten-specific B cells as important antigen presenting cells in celiac disease. Together with the finding that the hmAbs cross-react with different gliadin peptides, it suggests that the gluten-specific B-cells could take up and display many different T-cell epitopes and consequently get help from many distinct gluten-specific T cells which have been demonstrated to be important for generating B-cell responses^29^.

The dominant T-cell response in celiac disease is directed towards α-gliadin and ω-gliadin peptides^13^,^30^,^31^, while the B-cell response seems to be directed to y/ω-peptides^11^,^17^. In this study, we demonstrated a preferential hmAb-enrichment of fragments from y-gliadin, ω-gliadin and LMW glutenin proteins. Strikingly few fragments of α-gliadin proteins were identified in the pull-down samples. Epitopes of α-gliadin are important for T cells in celiac disease^13^. This discrepancy between the T-cell and B-cell response may reflect true differences, but we do not exclude that methodological factors contribute to the observed bias. Testing of synthetic peptides demonstrated that the abundance of the different peptides in the gliadin fractions affected which peptides were pulled down with the hmAbs. Further, the gel filtration fractions of the gliadin digest containing the highest molecular weight peptides were not used in the pull-down experiments to facilitate the identification of motifs recognized by the hmAbs. Thus, long peptide fragments like the α-gliadin 33mer, may to some extent have been excluded from these analyzes. Noteworthy is also the observation that several hmAbs enriched for peptides harboring the T-cell epitope DQ2.5-glia-ω1, which is similar to the DQ2.5-glia-α1 epitope and contain the B-cell epitope motif QPQQPFP. The DQ2.5-glia-ω1 epitope is also an immunodominant T-cell epitope^31^.

In summary, this study provides insights into the epitopes of the gluten proteome that are targeted by antibodies of IgA plasma cells in the celiac disease intestinal lesion. The majority of the antibodies prefer to bind long deamidated peptide fragments with multiple copies of shared motifs, suggesting that gluten-specific IgA^+^ plasma cells of different celiac disease patients recognize the same gluten B-cell epitopes. As the long fragments also contain many different T-cell epitopes, this will lead to generation of strong antibody responses by effective presentation of several distinct T-cell epitopes and establishment of T-cell help to B cells.

## Additional Information

**How to cite this article**: Dørum, S. *et al.* Gluten-specific antibodies of celiac disease gut plasma cells recognize long proteolytic fragments that typically harbor T-cell epitopes. *Sci. Rep.* **6**, 25565; doi: 10.1038/srep25565 (2016).

## Supplementary Material

Supplementary Figures

Supplementary Table 1

## Figures and Tables

**Table 1:** Overview of the gliadin-reactive hmAbs.

hmAb	Selecting antigen	PLQPEQPFP reactivity^5^	DA 33mer reactivity^5^	CT gliadin reactivity^5^	Binding DA peptides >> NA peptide***^5^	# MS identified hmAb enriched peptides	Longest common 7mer motif in hmAb enriched peptides (% peptides pre/post hmAb pull-down)	# DA peptides pre/post hmAb pull-down
1002-1E01	PLQPEQPFP	Yes	Yes	Yes	No	139	QPQQPFP (39%/94%)	48%/77%
1130-3B04	Deamidated 33-mer	Yes	Yes	Yes	No	257	QPQQPFP (22%/95%)	88%/ 98%
1130-3A02	Deamidated 33-mer	Yes	Yes	Yes	No	21	QPQQPFP (37%/100%)	33%/43%
1130-2A02	Heat/acid treated CT gliadin	Yes	Yes	Yes	No	222	QPQQPFP (21%/92%)	26%/85%
1002-1E03	PLQPEQPFP	Yes	No	Yes	Yes	381	QPQQPFP (35%/95%)	27%/85%
1114-1G01	PLQPEQPFP	Yes	Yes	Yes	Yes	22	X_1_QPQQPX_2_ (X_1_ = P, S; X_2_ = I, L, F) (3%/100%)	33%/96%
1130-3B01	Deamidated 33-mer	Yes	Yes	Yes	2–3 log	48	QPQQXFP (X = P, S, T, Q) (49%/98%)	48%/82%
1130-3A05	Deamidated 33-mer	Yes	Yes	Yes	Not tested	552	QPQQPFP (25%/88%)	74%/100%
1130-3B03	Deamidated 33-mer	No	Yes	Yes	Yes	19	No common motif (<30%)	41%/9%
1130-3G05	Deamidated 33-mer	No	Yes	Yes	Yes	117 *	No common motif (<30%)	30%/29%
1050-5B05	Heat/acid treated CT gliadin	Yes	No	Yes	Not tested	23	X_1_QPQQPX_2_ (X_1_ = Q P, I/L; X_2_ = F, Q, A) (30%/83%)	27%/35%
1065-4C01	Heat/acid treated CT gliadin	No	No	Yes	Not tested	4	-	-
1065-4G05**	Heat/acid treated CT gliadin	No	No	Yes	Yes	0	-	-

Patient number, isolation method (selecting antigen denotes the antigen used to isolate the IgA^+^ plasma cells of which the hmAb was cloned) and reactivity to antigens in ELISA and/or AlphaLISA are shown as well as the number of hmAb-enriched peptides identified by MS, the most frequent 7mer motif among these peptides and % deamidated (DA) peptides pre/post hmAb pull-down.

^*^31 peptides removed from the list as they were identified in the negative control sample.

^**^For this hmAb 28 μg and not 40 μg was used in pull-down experiment.

^***^Tested PLQPEQPFP/PLQPQQPFP for all hmAbs except for 3B03 and 3G05 where the DA and native α-gliadin 33mer was tested.

**Table 2:** Percentage of peptides pulled down with the eleven hmAbs that harbor known gluten T-cell epitopes.

T-cell epitope		Peptides (P) pulled down by hmAbs										
Name	Sequence	1002-1E01 (139 P)	1130-3B04 (257 P)	1130-3B01 (48 P)	1002-1E03 (381 P)	1130-3B03 (19 P)	1130-3G05 (117*P)	1130-3A02 (21 P)	1130-2A02 (222 P)	1114-1G01 (22 P)	1050-5B05 (23 P)	1130-3A05 (552 P)
*Peptides with any T-cell epitope*		*84%*	*91%*	*88%*	*85%*	*21%*	36%	86%	84%	14%	44%	73%
DQ2.5-glia-α1a	PFPQPQLPY	–	–	–	–	5%	1%	–	–	–	4%	–
DQ2.5-glia-α1b	PYPQPQLPY	–	–	–	–	–	1%	–	–	–	–	–
DQ2.5-glia-α2	PQPQLPYPQ	–	–	–	–	5%	1% (2x)	–	–	–	–	–
DQ2.5-glia-α3	FRPQQPYPQ	–	–	–	–	–	–	–	–	–	–	–
DQ2.5-glia-γ1	PQQSFPQQQ	–	0.5%	17%	0.5%	–	3%	–	0.5%	–	9%	7%
DQ2.5-glia-γ2	IQPQQPAQL	–	–	–	–	–	–	–	–	–	–	–
DQ2.5-glia-γ3	QQPQQPYPQ	1.5%		–	3% (1.1x)	–	–	5%	3%	–	–	2%
DQ2.5-glia-γ4a	SQPQQQFPQ	–	2%	–	1%	–	–	5%	–	–	–	–
DQ2.5–glia-γ4b	PQPQQQFPQ	0.5%	0.5%	23%	3%	–	2%	–	0.5%	–	–	3%
DQ2.5-glia-γ4c	QQPQQPFPQ	77% (1.7x)	84% (1.7x)	58% (1.2x)	79% (1.7x)	11%	27% (1.2x)	81% (1.5x)	74% (1.5x)	–	30% (1.1x)	63% (1.3x)
DQ2.5-glia-γ4d	PQPQQPFCQ	1%	–	–	–	–	–	–	0.5%	–	–	–
DQ2.5-glia-γ5	QQPFPQQPQ	58% (1.2x)	45% (1.2x)	31%	47% (1.2)	11% (1.5x)	9% (1.3x)	57% (1.3x)	60% (1.2x)	–	17%	28%
DQ2.5-glia-ω1/ DQ2.5-sec-1/ DQ2.5-hor-1	PFPQPQQPF	10%	7%	6%	7%	-	2%	29%	11%	14%	17%	5%
DQ2.5-glia-ω2	PQPQQPFPW	2%	5%	4%	3%	–	–	19%	5%	–	13%	4%
DQ2.5-glut-L1	PFSQQQQPV	0.5%	–	2%	0.5%	5%	4%	–	–	–	–	–
DQ2.5-glut-L2	FSQQQQSPF	–	–	–	–	–	–	–	–	–	–	–
DQ2.5-hor-2/ DQ2.5-sec-2	PQPQQPFPQ	1.5%	1%	2% (2x)	0.5%	–	–	–	1%	–	–	–

The average number of T-cell epitope per peptide is given in brackets.

^*^31 peptides removed from the list as they were identified in the negative control sample.
