# Supplementary material for: Gluten-specific antibodies of celiac disease gut plasma cells recognize long proteolytic fragments that typically harbor T-cell epitopes
Source: Sci Rep. 2016 May 5;6:25565. doi: 10.1038/srep25565 (PMC4857092; doi:10.1038/srep25565)
Supplement: Supplementary Figures [file srep25565-s1.pdf]

Gluten-specific antibodies of celiac disease gut plasma cells recognize long proteolytic fragments that typically harbor T-cell epitopes

Siri Dørum, Øyvind Steinsbø, Elin Bergseng, Magnus Ø. Arntzen, Gustavo A. de Souza[1] and Ludvig M. Sollid

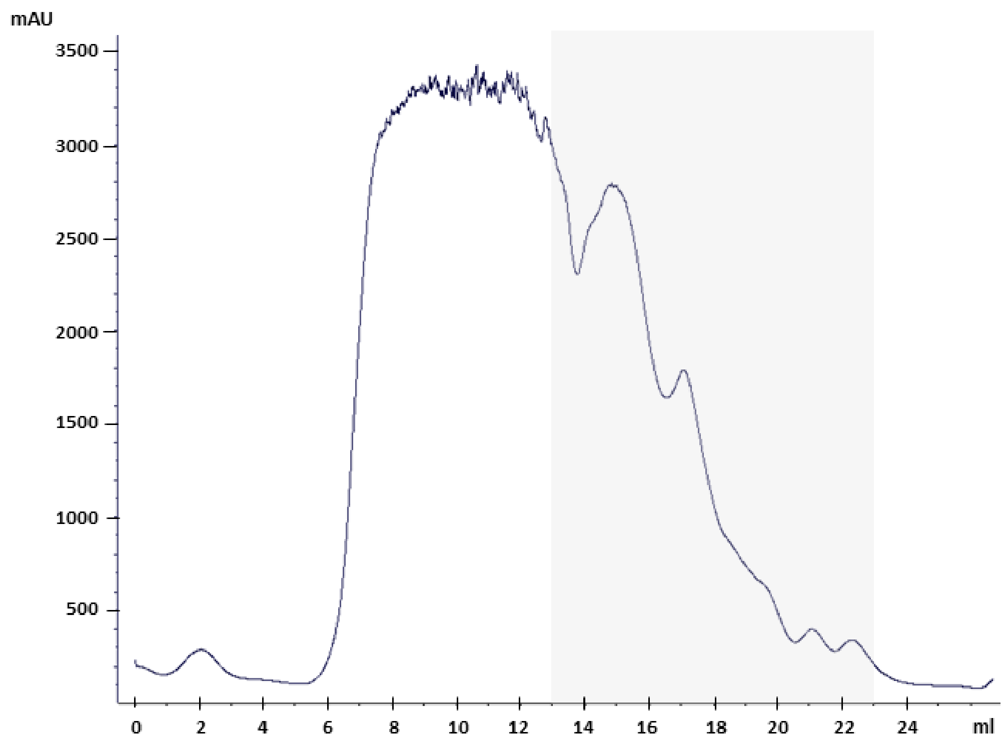

Supplementary Figure 1. Size exclusion chromatography of PTCEC gliadin. PTCEC gliadin was fractionated by gel filtration using a Superdex peptide 10/300 GL column. The fractionation was performed in two rounds and fractions marked in grey were selected for the enrichment procedure (representative chromatogram shown). The peptides were measured at absorbance 214 nm.

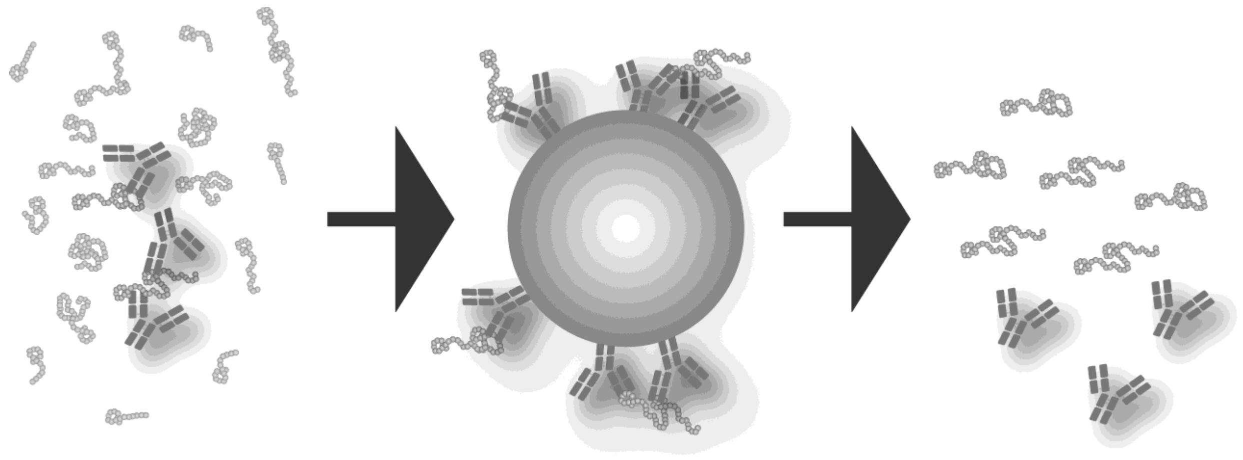

Supplementary Figure 2. Schematic view of the method for enrichment of peptide epitopes of gluten-specific hmAbs. The recombinant hmAbs were incubated with fractions of a TG2-treated digest of gluten, hmAb-peptide complexes were isolated by magnetic protein G beads and extensive washing was applied. The hmAb bound peptides were eluted by acidification and the eluted peptides were analysed by LC-MS/MS.

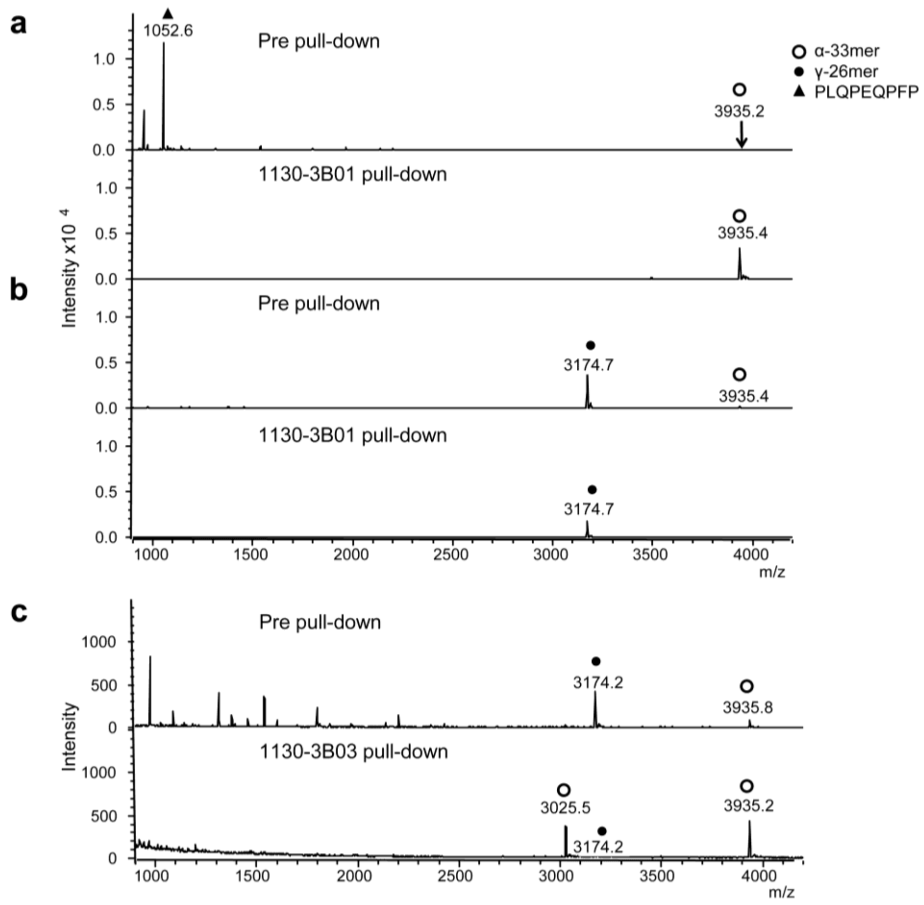

Supplementary Figure 3. Analysis of factors affecting peptide pull-down by MALDI-TOF. MALDI-TOF mass spectra of equimolar amounts of pairs of peptides pre and post pull down with the hmAbs 1130-3B01 and 1130-3B03. A and B) 1130-3B01 enrichment from the peptide pairs α-gliadin 33mer and PLQPEQPF peptide (A) or α-gliadin 33mer and γ-gliadin 26mer (B). C) 1130-3B03 enrichment from the peptide pair α-gliadin 33mer and γ-gliadin 26mer. In the lower panel of C, a peptide with the m/z value 3025.5, probably a shorter version of the α-gliadin 33mer peptide due to miss-coupling during peptide synthesis (Δ- m/z 888.5 = LLLLLPPQ/LLLPPPEQ, error 0.4 Da), is present. In the upper panel of C, the peptides in the lower mass range are fragments of the 33mer peptide generated by high laser power.
